# Distinguishing Gait Patterns in PD Patients Under Different Treatments via Recurrence Plots and Vision Transformer Fusion

**DOI:** 10.1109/OJEMB.2026.3667045

**Published:** 2026-02-23

**Authors:** Vasileios Skaramagkas, Georgios Karamanis, Iro Boura, Chariklia Chatzaki, Cleanthe Spanaki, Zinovia Kefalopoulou, Manolis Tsiknakis

**Affiliations:** Department of Electrical and Computer EngineeringHellenic Mediterranean University 112178 GR-710 04 Heraklion Greece; Institute of Computer ScienceFoundation for Research and Technology Hellas (FORTH) GR-700 13 Heraklion Greece; Department of NeurologyPatras University Hospital GR-264 04 Patras Greece; School of MedicineUniversity of Patras 37795 GR-265 04 Patras Greece; School of MedicineUniversity of Crete 37778 GR-710 03 Heraklion Greece; Department of Basic and Clinical Neuroscience, Institute of Psychiatry, Psychology and NeuroscienceKing's College London 4616 London WC2R 2LS U.K.; School of MedicineUniversity of Crete 37778 GR-710 03 Heraklion Greece; Department of NeurologyUniversity Hospital of Heraklion 37797 GR-715 00 Heraklion Greece

**Keywords:** Parkinson's disease, deep brain stimulation, gait analysis, vision transformers, generative adversarial networks

## Abstract

*Goal:* This study aims to develop an innovative gait analysis framework using recurrence plots (RPs) to differentiate gait patterns between Parkinson's disease (PD) patients under varying treatment regimes and healthy individuals. *Methods:* Pressure sensor data were transformed into RPs and analyzed using a Vision Transformer (ViT) model with multiple fusion strategies. To address class imbalance, a conditional Deep Convolutional Generative Adversarial Network (DC-GAN) was employed to generate synthetic gait data. Four ViT-based fusion architectures were investigated and evaluated across multi-class and binary classification tasks. *Results:* The dual ViT stream with late fusion achieved the highest accuracy in multi-class classification (94.58%), while the cross-attention fusion model outperformed others in binary classification tasks. *Conclusions:* The findings indicate that gait characteristics captured via RPs can effectively distinguish between PD patients under different treatments and healthy controls. This approach provides a data-driven pathway for objective and individualized assessment of PD therapies, potentially supporting improved clinical decision-making.

## I. Introduction

Parkinson's disease (PD) is a prevalent, chronic neurodegenerative disorder, impacting 0.1-0.2% of the general population [1]. The rate escalates tenfold post-60 years of age, with a significant increase in the global population of affected individuals in recent years [2]. This amount is anticipated to increase further in the forthcoming decades, presenting substantial problems for healthcare providers and policymakers worldwide [3]. PD is clinically defined by bradykinesia and a minimum of one other primary motor symptom, such as rigidity or resting tremor, in conjunction with supplementary supporting and excluding characteristics [4]. Additional motor characteristics of the disease include diminished facial ex- pression, micrographia (decreasing handwriting size), speech impairments, walking difficulties, and dysphagia. These symptoms increasingly disrupt patients' daily activities, profoundly affecting their quality of life [5]. As motor dysfunction progresses, gait irregularities deteriorate and postural instability emerges, posing a continuous and growing risk on patients' autonomy and safety.

In lack of a neuroprotective treatment, the management of PD is currently focused on adequate symptomatic control. Pharmacological dopaminergic substitution provides stable improvement in cardinal motor symptoms, especially during early stages. In later stages, when motor fluctuations and dyskinesias develop, more advanced and invasive options, such as deep brain stimulation (DBS), should be employed [6]. DBS is an established neurosurgical treatment for PD, based on the stereotactic implantation of electrodes in specific brain loci and neuromodulation of targeted areas. Notably, the response of parkinsonian symptoms to available treatments, including DBS, can vary substantially among individuals. In particular, the effects of DBS on gait and balance remains an area of active investigation, underscoring the multifactorial origins of gait disturbances in PD [7].

Gait impairment in PD develops progressively, exhibiting complex patterns of gait irregularities as the condition ad- vances. These encompass lowered locomotion smoothness, heightened interlimb asymmetry [8], reduced speed, abbreviated step lengths [9], shuffling gait, prolonged double-limb support, disjointed turns, and impaired balance and postural control [10]. Gait-related data are utilized to forecast fall hazards, which considerably impact the quality of life for people with PD [11]. Beyond discrete gait abnormalities, gait variability has emerged as a particularly strong predictor of fall risk, with increased stride-to-stride fluctuations shown to prospectively identify future fallers in both community-dwelling older adults and patients with PD [12], [13]. Falls in advanced PD are frequently associated with a paroxysmal motor phenomenon known as “Freezing of Gait” (FoG), defined as a short, sudden episode where a person in unable to move their feet forward despite intending to walk [14]. More than 60% of PD sufferers encounter some variant of freezing as the disease progresses; yet, the inadequate comprehension of its pathophysiology and circuit mechanics obstructs successful treatment [15]. Recent extensive studies have shown that wearable sensor data, when integrated with machine learning techniques, may accurately identify FoG episodes and reveal clinically significant patterns, encompassing contextual and temporal influences [16]. Furthermore, research indicates that gait features, such as step duration, step length, double support time, and swing time, can be utilized to develop predictive models for FoG episodes [17]. Consequently, an objective, quantitative evaluation of gait could improve existing methodologies assessing diagnosis and monitoring of symptoms, therapeutic management, rehabilitation, and fall risk for PD patients, including those undergoing DBS.

Gait analysis is essential for controlling and monitoring the course of PD symptoms [9]. Wearable sensor-equipped systems have been created to record several motor symptoms, including gait irregularities, balance difficulties, and tremors, which cannot be entirely captured through clinical observations alone [18]. The rationale behind wearable devices lies in their potential to characterize motor symptoms in PD. The National Institute for Health and Care Excellence (NICE) has conditionally endorsed many devices for the remote monitoring of PD, including Kinesia 360, KinesiaU, PDMonitor, Personal KinetiGraph (PKG), and STATON [19]. Studies indicate that not only gait patterns can differentiate PD patients from healthy persons [20], [21], but there is additionally an increasing interest in utilizing machine learning and deep learning (DL) methodologies that leverage sensor data (e.g., accelerometers, gyroscopes, pressure sensors) and/or video recordings to monitor and fore- cast the progression of PD [22]. Video-based motion analysis methods necessitate expensive equipment and are limited to controlled indoor situations, whereas wearable sensors provide a more economical option, facilitating long-term monitoring in real-world settings [23].

Numerous studies utilizing wearable data have concentrated on examining gait in PD through DL methodologies, especially for detecting FoG occurrences, which are generally difficult to evaluate manually. Conventional techniques for FoG identification necessitate laborious expert annotations; how- ever, recent innovations provide automated alternatives. The Convolutional 3D Attention Network (C3DAN) was created to measure FoG, utilizing video data with an accuracy of 79.30% [24]. Hybrid networks that integrate convolutional neural network (CNN) and long short term memory (LSTM) architectures have demonstrated potential, exemplified by a system created by Thu et al. achieving 90.01% accuracy [25]. Furthermore, acceleration signals and spectrograms obtained from PD patients were utilized in a DeepCNN-LSTM model to identify FoG with an accuracy of 94.30% [26].Additional research employed a SE-CNN to enhance FoG detection, attaining an accuracy of 95.66% [27]. Researchers have also investigated vision-based FoG detection through graph sequence modeling by implementing a graph sequence recurrent neural network (GS-RNN) which exhibited commendable efficacy in FoG identification, achieving AUC of 0.80 [28], while Naghavi et al. utilized transfer learning to enhance FoG detection, attaining 87.40% detection rate [29]. Finally, in 2025, researchers proposed a DL-based method for detecting FoG in PD using a novel convolution bottleneck attention–BiLSTM (CBA-BiLSTM) architecture. While they reported a striking 99.88% accuracy—achieved by reducing computational complexity through ensemble channel selection and attention mapping for real-time monitoring—such near-perfect performance warrants cautious interpretation, especially given the challenges of generalization and real-world deployment [30].

Moreover, various DL methodologies seek to categorize PD patients according to the severity of motor symptoms, focusing on gait analysis. Yang et al. introduced the PD-ResNet architecture for classifying PD severity according to the H&Y scale, attaining an accuracy of 92% [31]. Ogul et al. employed a Siamese Recurrent Network for classifying gait data according to the UPDRS scale, attaining an accuracy of 81% [32]. One year later, Guo et al. designed a spatial-temporal attention graph convolutional network for automated gait analysis, attaining an accuracy of 98.90% in classifying PD severity [33]. These findings underscore the promise of wearable sensor-based systems integrated with machine learning for the monitoring and management of PD.

Despite extensive research employing wearable sensors for gait analysis and the classification of PD symptoms, the influence of various treatments on gait patterns remains inadequately investigated within the framework of automated categorization. Considering that DBS can profoundly improve motor function, yet its impact on gait is still controversial, a systematic and data-oriented method for categorizing DBS and non-DBS circumstances may yield critical insights into treatment outcomes [34].

This work introduces a novel framework for distinguishing PD patients under different treatments and healthy controls. Using recurrence plots (RPs) [35] derived from insole pressure data collected under a standardized gait protocol, we move beyond prior studies that focused mainly on binary PD classification or severity scoring. Our approach specifically addresses DBS-related categorization by exploiting RPs as high-dimensional features to capture subtle gait changes induced by therapeutic interventions. To this end, we apply Vision Transformers (ViTs) and systematically compare multiple fusion strategies across both multiclass and binary contexts. Data augmentation is further supported through a conditional Deep Convolutional GAN (DC–GAN), whose generated samples were validated with multiple metrics to ensure robustness. Together, these methods provide a data-driven pathway toward objective treatment-specific gait signatures and lay the groundwork for individualized monitoring of PD motor symptoms and refined gait-based biomarkers, including

## II. Materials and Methods

This section offers a succinct summary of the study design, participants, preprocessing, and modeling approach. Comprehensive technical details—including comprehensive sensor specifications, annotation protocols, data preprocessing procedures, the whole DC-GAN and Vision Transformer architectures, training setups, and extensive validation metrics—are available in the Supplementary Materials.

### A. Data Collection and Experimental Procedure

#### 1) Study Participants

A total of 211 subjects completed the protocol: 167 PD patients (61 female; mean age $65.2 \pm 9.57$; mean LEDD $628.1 \pm 382.6$) and 44 healthy controls (mean age $54.6 \pm 17.12$). Among PD patients, 154 received dopaminergic therapy only and 13 had undergone DBS. For analysis, we defined three groups: A (controls, $n=44$), B (DBS, $n=13$), and C (non-DBS, $n=154$). Ethical approval and informed consent were obtained from all participants (11692/19-05-2023, 347/13-07-2023, 9/01-04-2020).

#### 2) Experimental Protocol

Gait was assessed using the Smart-Insole protocol [21], including the Walk Straight and Turn (WST) and modified Timed Up and Go (mTUG) tests. Subjects wore pressure-sensor insoles (Moticon, Germany), which have been previously validated for plantar pressure and temporal gait analysis in overground walking [9], [21]. Recordings were performed during ON-medication state for PD patients and ON-stimulation for DBS patients.

### B. Data Preprocessing

**Fig. 1.**
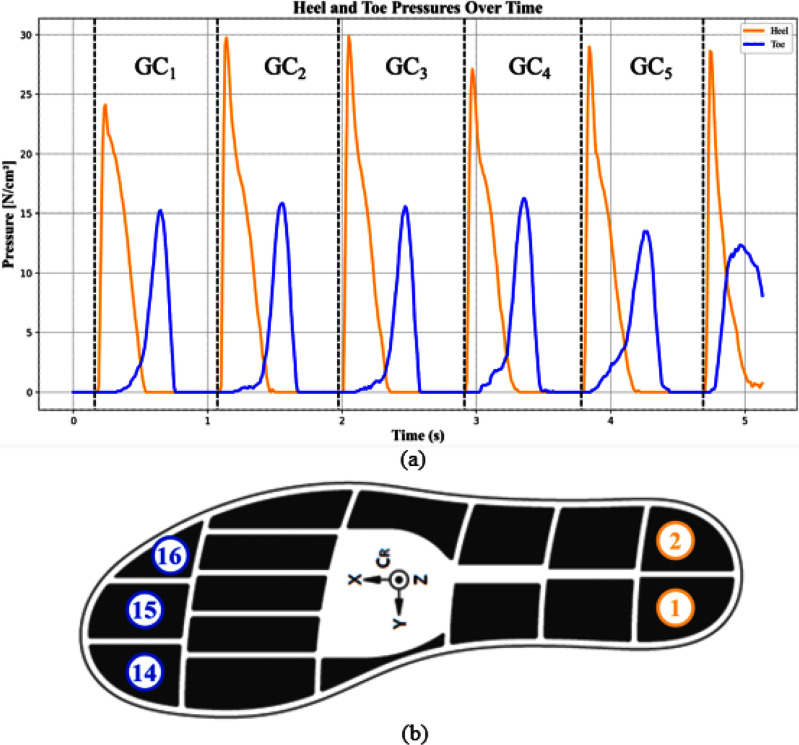
(a) Indicative sample of heel and toe pressure values over time and illustration of gait cycle segmentation using heel-strike events. (b) Layout of the pressure-sensor insole, where the numbered regions correspond to individual pressure sensor elements embedded in the insole (sensors 1,2: heel region; sensors 14–16: toe region).

Gait cycles were segmented using heel-strike events, normalized to one second duration, and reduced to heel and toe pressure channels (Fig. 1). Non-walking segments (turning, sitting) were excluded.

### C. Synthetic Data Generation

Synthetic data generation was performed in a fold-wise manner to prevent data leakage. For each cross-validation fold, the DC-GAN was trained exclusively on the training subset, and synthetic samples were used only to augment training data. To address class imbalance, we implemented a conditional Deep Convolutional GAN (DC-GAN) conditioned on subject ID and treatment group. The model generated synthetic gait cycles that preserved distributional properties of the original dataset. The validation of synthetic data was conducted by calculating several statistical and distance-based metrics to assess the resemblance between synthetic and original data as well as by extracting quantitative metrics (see Supplementary Material).

### D. Recurrence Plots (RPs)

**Fig. 2.**
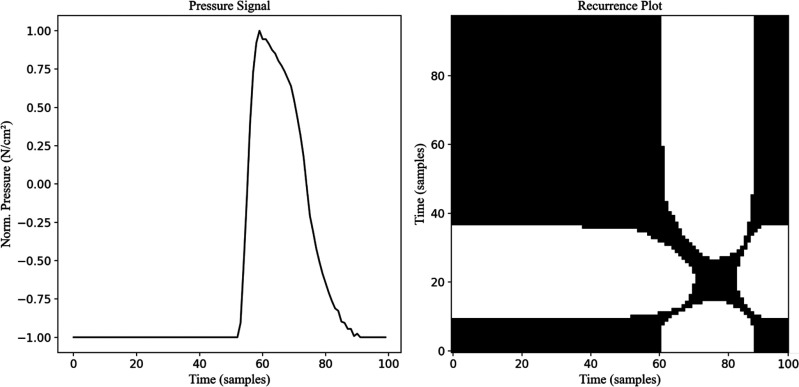
Pressure values during one randomly selected gait cycle (left) and the respective RP computed (right).

Heel and toe pressure signals were transformed into recurrence plots to capture temporal dynamics [36]. Consequently, RPs were generated from the real and synthetic data to examine the dynamic behavior of heel and toe pressure signals, as seen in Fig. 2.

### E. Vision Transformer (ViT) Classification

We explored four ViT-based fusion strategies to classify treatment groups from RPs: (1) Dual-stream with late fusion, (2) Attention-based feature fusion, (3) Cross-attention, and (4) Ensemble learning. A concise overview is presented in the Results, while model architectures and implementation details are described in the Supplementary Materials.

## III. Results

### A. Conditional DC-GAN Gait Generation Validation

#### 1) Original and Synthetic Data

To evaluate the performance of the DC-GAN, synthetic data generation was guided by two key principles. First, the number of synthetic gait cycles generated per subject matched that of the original dataset (see Table I), thereby preserving the intra-subject distribution. Second, the overall volume of generated data was designed to reflect the class distribution of the three groups as observed in the original dataset. Given that DBS patients comprised a minority of the dataset, Group B was augmented by generating synthetic samples at 10 times the original size, while Group A (controls) was upsampled by 2 times. This balancing strategy ensures that the model can learn treatment-specific gait features without being biased toward majority classes. Crucially, this step supports our broader clinical goal: enabling the model to recognize distinct gait patterns associated with therapeutic interventions, rather than simply reflecting dataset imbalances.

**TABLE I:** Gait Cycles for Groups A,b, and C in the Original and Synthetic Datasets

**Type**	**Control (A)**	**DBS (B)**	**non-DBS (C)**
Original	7,517	2,207	25,600
Synthetic (eval.)	7,517	2,207	25,600
Synthetic (DL)	22,551	24,277	-

#### 2) Performance Metrics

The results of the evaluation of the synthetic dataset using performance metrics are summarized in Table II. The assessment of the synthetic dataset through several performance metrics indicates a significant resemblance between the actual and created data. Specifically, the Mean Absolute Error (MAE) and Mean Squared Error (MSE) values consistently exhibit low levels across all conditions, signifying negligible divergence between synthetic and actual data. The cosine similarity values indicate that the generated data exhibits a similar angular alignment to the original dataset, with heel region values somewhat above those of the toe region. The Kullback-Leibler (KL) and Jensen-Shannon (JS) divergence measures indicate that the probability distributions of the synthetic dataset closely mirror those of the real data, exhibiting slight differences between the three groups. Moreover, Wasserstein Distance (WD) and Fréchet Inception Distance (FID) are both minimal, indicating that the statistical properties of the generated data closely match those of the real samples. These findings validate the synthetic data's utility for training robust models capable of capturing clinically relevant treatment differences. Notably though, group B demonstrates slightly elevated divergence values, especially in the toe region, which may suggest minor differences in foot pressure dynamics for this group.

**TABLE II:** Performance Metrics of the Implemented Conditional DC-GAN (All Metrics are Unitless, Computed on Normalized Gait Cycle Data)

**Foot region**	**MAE**	**MSE**	**CS**	**KL**	**JS**	**WD**	**FID**
	0.27	0.14	0.63	2.03	0.22	0.04	0.04
	0.26	0.14	0.60	1.94	0.25	0.03	0.03
	0.27	0.15	0.59	2.03	0.25	0.03	0.03
	0.28	0.16	0.60	2.00	0.24	0.04	0.04
	0.26	0.16	0.59	2.09	0.25	0.12	0.12
	0.26	0.16	0.60	2.12	0.24	0.08	0.08
	0.27	0.17	0.60	2.16	0.24	0.05	0.05
	0.27	0.17	0.62	2.18	0.24	0.02	0.02
	0.27	0.15	0.55	2.19	0.24	0.12	0.12
	0.23	0.13	0.53	2.04	0.27	0.04	0.04
	0.24	0.13	0.52	2.07	0.27	0.05	0.05
	0.25	0.13	0.53	2.18	0.26	0.06	0.06
	0.24	0.14	0.57	1.99	0.25	0.03	0.03
	0.25	0.14	0.56	2.30	0.26	0.03	0.03
	0.26	0.15	0.56	2.37	0.25	0.01	0.01
	0.26	0.15	0.55	2.49	0.26	0.02	0.02

^0^MAE: Mean Absolute Error; MSE: Mean Squared Error; CS: Cosine Similarity; KL: Kullback–Leibler Divergence; JS: Jensen–Shannon Divergence; WD: Wasserstein Distance; FID: Fréchet Inception Distance.

#### 3) Gait Parameters Comparison

**Fig. 3.**
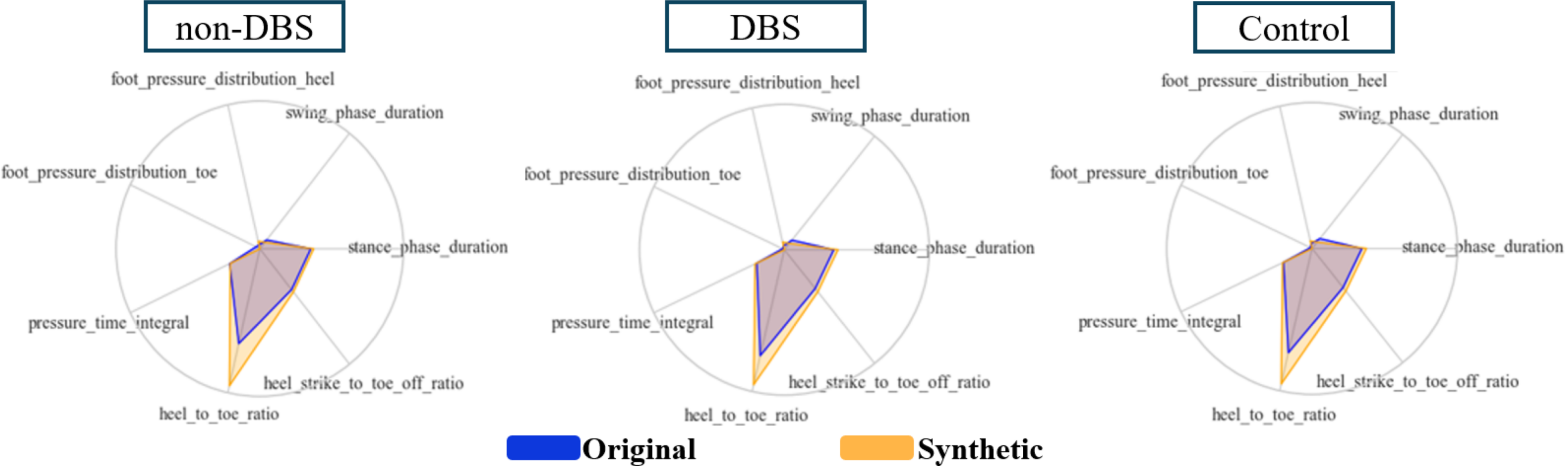
Radar plots comparing the original and synthetic data. Each plot represents a different group (DBS, non-DBS, Control). The black area indicates the original data, while the orange area represents the generated ones.

Fig. 3 presents radar plot comparisons of gait metrics derived from original and synthetic samples across the three participant groups. Across all categories, the synthetic data exhibit a slightly extended stance phase duration, accompanied by a proportionally reduced swing phase, suggesting subtle alterations in temporal gait dynamics. Foot pressure distribution in the synthetic data shows a modest increase in the heel region and a corresponding decrease in the toe region, reflecting a minor imbalance in pressure allocation. Additionally, the heel-to-toe ratio is slightly elevated, implying a potentially altered weight-shift pattern during gait. Importantly, the pressure-time integral remains consistent between original and synthetic datasets, indicating that the overall pressure exertion patterns are well preserved. Despite these minor deviations, the synthetic data closely approximates the original, demonstrating the DC-GAN's effectiveness in capturing essential gait characteristics while introducing physiologically plausible variability.

### B. Classification

For the classification experiments, A balanced version of our dataset comprising of original gait cycles from group C and a combination of original and synthetic gait cycles from the other two groups was used, as described in Table I, all transformed into grayscale RPs. The RPs were later resized into $72 \times 72$ and fed in the four models as input. After multiple experiments with different sizes, $72 \times 72$ was determined as the optimal image size both in terms of classification performance and memory efficiency. This classification framework is intended not merely to distinguish between subject groups, but to serve as a potential mechanism for identifying treatment-specific gait signatures.

Every input image is subjected to data augmentation, encompassing random flipping, rotation, zooming, and contrast modifications. The model retrieves 72× 72 patches from each image, where each patch is of size 6× 6, leading to 144 patches per image in total. The patch embeddings undergo processing through eight transformer levels, each comprising multi-head self-attention (four heads) and feed-forward layers utilizing GELU activation. Each image patch is embedded into a 64-dimensional vector, while the transformer layers consist of 128 and 64 neurons. Features extracted from both heel and toe images are fused using the four strategies detailed in the Supplementary Material. The fused features are then passed through a final processing block that enhances representation using multihead self-attention, one-dimensional convolutional layers (1D CNNs with 1024 and 512 filters), and fully connected layers with GELU activation, batch normalization, and residual connections. Finally, a classification layer outputs the logits corresponding to the target classes.

#### 1) Multi-Class Classification

**Fig. 4.**
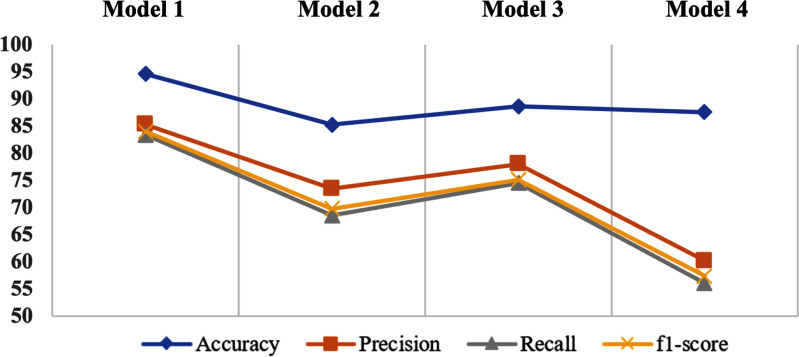
Performance metrics for the four different implemented ViT models for the multi-class classification task.

Fig. 4 illustrates the performance metrics for the four ViT models utilized in the multi-class classification task differentiating between three groups; Control, non-DBS, and DBS patients. Model 1 attains the highest overall accuracy of 94.58%. It exhibits high precision (85.31%) and recall (83.42%), resulting in the superior f1-score (83.99%) compared to all models. This indicates that late fusion is proficient in utilizing complementary information from various feature streams to enhance classification performance. Model 2 demonstrates a decline in performance, achieving an accuracy of 85.29%. The precision (73.46%), recall (68.55%), and f1-score (69.77%) are markedly inferior than those of Model 1. Similarly, Models 3 and 4 attain accuracies 88.61% and 87.59%, respectively, slightly surpassing Model 2. The markedly diminished precision and recall of the last 3 models indicate that they encounter difficulties in generalizing effectively across the three groups.

#### 2) Binary Classification

Table III delineates the performance metrics of the four executed ViT models for the binary classification tasks. Specifically, model 1 attains superior accuracy in all binary classifications, exhibiting optimal performance in differentiating between groups B and C, achieving an accuracy 99.26%. The classification demonstrates robust predictive power with precision 91.73%, recall 91.35%, and f1-score 91.53%. The model demonstrates efficacy in distinguishing A from C, with accuracy 96.54% and f1-score 84.87%. Nonetheless, the f1-score for differentiating A from B is marginally reduced, indicating a comparatively elevated misclassification rate between these two cohorts.

**TABLE III:** Performance Metrics for the Four Implemented ViT Models in Binary Classification Tasks. Class Labels Correspond To: A = Healthy Controls, B = DBS-Treated PD Patients, C = non-DBS PD Patients.

**Model**	**Classes**	**Acc.**	**Pr.**	**Rec.**	**f1**
Model 1 Dual ViT Stream with Late Fusion	A, B	96.55	81.25	79.52	80.20
	A, C	96.54	85.84	84.11	84.87
	B. C	99.26	91.73	91.35	91.53
Model 2 Feature Fusion via Attention Mechanism	A, B	92.01	58.93	54.93	56.59
	A, C	97.83	83.82	82.73	83.22
	B. C	99.71	94.24	94.10	94.17
Model 3 Cross Attention Between Heel and Toe Features	A, B	98.88	96.61	95.05	95.39
	A, C	99.16	95.48	95.07	95.16
	B. C	99.64	95.76	95.58	95.66
Model 4 Separate Models Training and Ensemble Learning	A, B	97.2	75.89	74.49	75.15
	A, C	97.1	74.84	73.39	74.04
	B. C	99.45	90.43	90.16	90.29

Model 2 exhibits a significant decline in performance when differentiating between A and B patients, attaining the lowest f1-score. However, its classification of B compared to C groups is more reliable, with accuracy 99.71%. The distinction between A and C remains robust, with f1-score of 83.22%. Model 3 exhibits the most reliable performance across all binary classification tests. It attains exceptional accuracy across all group comparisons, achieving 98.88% for A versus B, 99.16% for A versus C, and 99.64% B versus C. The f1-scores are elevated, varying from 95.16% to 95.66%, signifying a balanced precision and recall. In addition, model 4 achieves high accuracy across all tasks, exhibiting percentages marginally inferior to those of model 3. Nevertheless, the f1-scores for differentiating A from B and A from C are significantly reduced. Overall, the results demonstrate that model 3 attains the superior classification performance in binary tasks, exhibiting consistently elevated precision, recall, and f1-scores across all group comparisons.

#### 3) System Specifications

Experiments were conducted on a high-performance GPU cluster using 8 NVIDIA A100-SXM4-40 GB GPUs per node, AMD EPYC 7742 64-core CPUs (up to 2.25 GHz), and 1 TiB RAM. Models were trained with TensorFlow-GPU 2.5.0 (Keras API), using AdamW (initial learning rate $10^{-4}$, weight decay), binary cross-entropy loss, and validation accuracy for model selection. Training used a batch size of 128 for 50 epochs, consuming 38 GB GPU memory and taking 30–35 minutes per run.

## IV. Discussion

The conditional DC–GAN generated realistic synthetic gait cycles for all groups, closely aligning with real data based on various statistical and distributional metrics. Minor discrepancies were noted mainly in DBS toe-pressure dynamics; these are minimal in scale and may indicate treatment-specific gait mechanics rather than model aberrations, hence endorsing the utilization of GAN-augmented data for class balance without sacrificing physiological plausibility. Furthermore, aggregate gait metrics comparing real and synthetic data exhibited minor, consistent variations (e.g., marginally extended stance and diminished swing) while maintaining overall pressure-time profiles, thereby affirming that the generated samples encapsulate fundamental gait characteristics beneficial for subsequent learning.

Vision Transformer (ViT) models shown high accuracy in identifying treatment-specific patterns across classifiers. In the three-class framework (Control, non-DBS, DBS), the dual-stream ViT with late fusion achieved the highest accuracy of 94.58%, demonstrating that the acquisition of region-specific representations and subsequent late fusion results in optimal group differentiation. In binary comparisons, the cross-attention ViT consistently outperformed others, indicating that explicit heel-toe interactions offer a more nuanced background for group discrimination. The dual-stream late-fusion model shown strong performance, especially in distinguishing between DBS and non-DBS, while attention-based feature fusion and simple ensembling proved to be less dependable. Differentiating DBS from controls proved to be more challenging, aligning with the tendency of DBS to modify gait towards healthy patterns. In general, integrative and physiologically informed fusion techniques seem superior for modeling intricate treatment effects. Comprehensive studies, including complete GAN validation metrics, comparisons of stance/swing and pressure distribution, and classification results for each model, are included in the Supplementary Materials.

From a clinical standpoint, the models were able to distinguish the three groups with high accuracy, suggesting that they recognize distinct gait patterns within subjects who could be considered as “similar”. In clinical as well as research settings, such DL models could facilitate a refined evaluation of the influence of various treatments on gait in PD patients, thus allowing more dynamic and personalized treatment approaches. Interestingly, the model's difficulty in distinguishing particularly DBS treated patients from controls, indicates that DBS may achieve a more normal gait pattern than oral medication [10]. With the emergence of innovative DBS techniques, adjusting stimulation in real time based on fluctuating clinical needs (i.e. adaptive DBS), the development of reliable biomarkers based on wearable sensor data, is increasingly becoming more crucial.

While the proposed ViT models show strong potential for distinguishing treatment-specific gait patterns, several limitations should be noted. First, their complexity and computational demands challenge real-time or portable use. Second, although the conditional DC–GAN reduced class imbalance, small discrepancies remained (particularly in DBS toe-pressure distributions), which could introduce subtle biases. Clinically, generalizability is uncertain since the cohort was recruited from a single national population. DBS patients were somewhat overrepresented, and subgroup sizes were limited. Assessments were performed in the ON-medication state, which did not always coincide with each patient's optimal response, and some advanced patients exhibited dyskinesias that may have influenced gait recordings. These factors may partly explain the variability observed in certain binary classification tasks. Extended discussion of these limitations is provided in the Supplementary Materials.

## V. Conclusion

This study presents a deep learning framework that employs gait analysis through recurrence plots and vision transformers to distinguish PD patients undergoing different treatments from healthy controls, exhibiting robust classification efficacy, especially with a dual ViT stream and cross-attention mechanisms. A key finding was the reduced separability between DBS- treated patients from healthy individuals, suggesting DBS may partially normalize some gait features, also highlighting the multifactorial nature of gait impairment in PD. Overall, our results indicate that machine-learning–based gait analysis may provide complementary, objective motor metrics that could ultimately support DBS programming and personalized treatment optimization, particularly in the context of emerging DBS technologies. Future endeavors will concentrate on investigating treatment-specific gait characteristics, implementing edge computing for real-time surveillance, and creating lightweight, AI-driven instruments to facilitate remote, cost-effective, and personalized PD therapy management.

## Supplementary Materials

Supplementary Materials
