# Supplementary material for: Distinguishing Gait Patterns in PD Patients Under Different Treatments via Recurrence Plots and Vision Transformer Fusion
Source: IEEE Open J Eng Med Biol. 2026 Feb 23;7:63–9. doi: 10.1109/OJEMB.2026.3667045 (PMC13068120; doi:10.1109/OJEMB.2026.3667045)
Supplement: Supplementary Materials [file supp1-3667045.pdf]

Distinguishing Gait Patterns in PD Patients under Different Treatments via Recurrence Plots and Vision Transformer Fusion (Supplementary Material)

Vasileios Skaramagkas[1], Georgios Karamanis[2], Iro Boura[3,4], Chariklia Chatzaki[1], Cleanthe Spanaki[3,5], Zinovia Kefalopoulou[2], and Manolis Tsiknakis[1]

I. MATERIALS AND METHODS

A. Data Collection and Experimental Procedure

1) Study participants: A total of 211 subjects were included and completed the protocol. Among them, 167 were PD patients with optimized symptom control (61 female; mean age; mean age 65.2 ± 9.57; mean Levodopa Equivalent Daily Dose 628.1 ± 382.6), while the rest of the participants include 44 healthy controls (34 males and 10 females) with a mean age 54.6 ± 17.12 were also enrolled. Among patients with PD, 154 were under dopaminergic substitution only, and 13 patients have undergone DBS.

Patients underwent protocol assessments both before and following medication administration (ON- and OFF-medication states, respectively). Healthy controls had a single assessment. DBS patients were assessed, while ON-stimulation which has been set at the clinically optimal electrical parameters. With a scope to assess gait patterns which resemble patients' realistic every day conditions, and compare it with normal gait, clinical and data from sensors were collected and analyzed from the ON-medication state (n=167). OFF-medication condition (after a 12-hour withdrawal of dopaminergic medication) is a commonly used state in research, but it represents an artificial condition with patients deprived of their standard treatment for long. ON-medication state mirrors best the desirable clinical condition of the patients and ideally the state they spend most of their time during a day. This focus enables us to characterize treatment-specific gait dynamics, helping to develop objective tools for assessing the real-life motor impact of various PD therapies.

[1]V. Skaramagkas, C. Chatzaki and M. Tsiknakis are with the Dept. of Electrical and Computer Engineering, Hellenic Mediterranean University, GR-710 04 Heraklion, Greece and the Institute of Computer Science, Foundation for Research and Technology Hellas (FORTH), GR-700 13 Heraklion, Greece vskaramag@ics.forth.gr

[2] G. Karamanis and Z. Kefalopoulou are with the Dept. of Neurology, Patras University Hospital, GR-264 04 Patras, Greece and the School of Medicine, University of Patras, GR-265 04 Patras, Greece

[3]I. Boura and C. Spanaki are with the School of Medicine, University of Crete, GR-710 03 Heraklion, Greece

[4]I. Boura is with the Dept. of Basic and Clinical Neuroscience, Institute of Psychiatry, Psychology and Neuroscience, King's College London, London WC2R 2LS, UK

[5]C. Spanaki is with the Dept. of Neurology, University Hospital of Heraklion, GR-715 00 Heraklion, Greece

2) Inclusion criteria: Healthy controls were enrolled at the Hellenic Mediterranean University (HMU), Crete, Greece during the Smart-Insole Study in 2020 [1]. PD participants were recruited during one year (June 2023 to May 2024) from the Movement Disorders Outpatient Clinics of two hospitals in Greece: the University General Hospital of Heraklion in Crete (UGHH) and the University General Hospital of Patra (UGHP). They fulfilled the Movement Disorder Society (MDS) criteria for Clinically Probable Parkinson's Disease [2]. Eligible patients were adults capable of independent ambulation, either unaided or with assistive devices, such as a cane or walking stick, classified within H&Y stages 1-4 during their ON-medication condition (for PD patients). Participants with confounding comorbidities, such as dementia or cognitive impairment, significant psychiatric diseases (e.g., major depression), profound auditory or visual impairment, and severe musculoskeletal issues that could hinder protocol adherence were excluded. Informed permission in writing was acquired from all subjects. All the clinical study protocols are approved by the Ethics Committees of both hospitals (11692/19-05-2023 for UGHH and 347/13-07-2023 for UGHP) and of HMU (9/01-04-2020).

For analysis purposes, we classified the participants of our study into three main sub- categories, Group A, Group B and Group C. Healthy controls appertain to Group A (n=44). Patients with DBS are included in Group B (n=13), while PD patients that have not undergone DBS belong to Group C (n=154). That includes patients with DA monotherapy (n=16), patients that are treated with Levodopa per os (n=135), and patients with LCIG (n=6).

3) Experimental Process: In this study, participants executed a segment of the Smart-Insole Gait Assessment Protocol [1], specifically the Walk Straight and Turn (WST) test and the modified Timed Up and Go (mTUG) test, while wearing insoles equipped with pressure sensors. As observed in Fig. 1, the WST test begun by commencing from an upright stance, proceed in a linear path for 10 meters, then reverse direction and return to the initial position. mTUG test begun from a sitting position, proceed in a linear path for 10 meters, then reverse direction and return to the initial sitting position [3]. During the initial phase of the mTUG test, participants are

required to cross their arms across their chest and rise up from a chair. Upon successfully standing, participants are instructed to walk a 10-meter distance, execute a turn, return along the same path, and resume their seated posture. The tests are conducted twice, at a normal walking speed. Gait research at various speeds has demonstrated that gait features are markedly influenced and can yield helpful insights for distinguishing between various groups of PD patients [3]–[5].

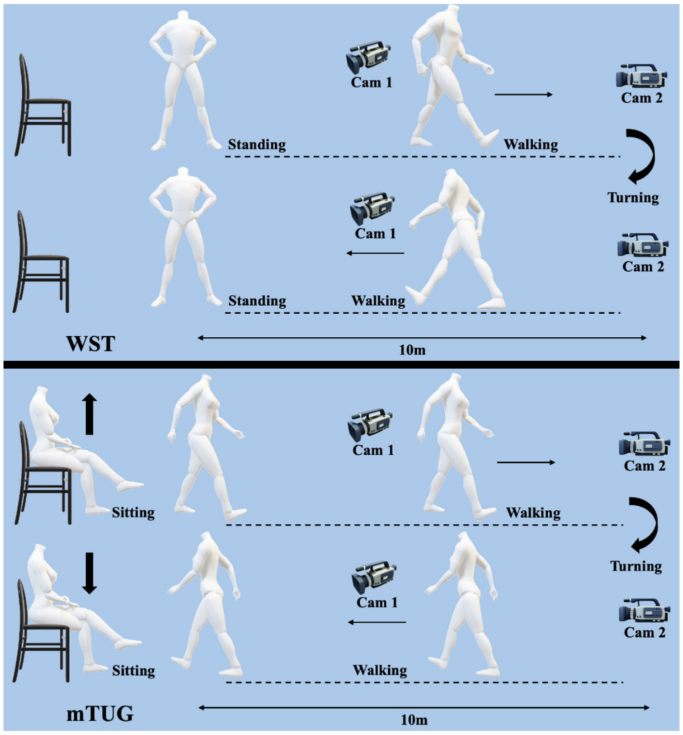

Fig. 1. Graphical representation of WST and mTUG tests.

4) Experimental Setup: For pressure data collection we used a validated sensor insole system, namely, the Moticon SCIENCE [6]. The insole system, comprises 16 capacitive pressure sensors, a 6-Axes Inertial Measurement Unit (IMU) providing acceleration and angular rate data, as well as components for power supply storage and data transmission integrated within the insole. A sampling rate of 100 Hz was utilized for the recordings. Each created file contains the date (ms), the pressure readings from 16 sensors (N/cm^2), the acceleration in the x, y, and z axes (g), the angular rates in ω_x, ω_y, and ω_z (dps), as well as the total force (N) and center of pressure coordinates in the x and y dimensions, as estimated by Moticon. To prevent discrepancies in the material configuration, lightweight and flexible pairs of shoes were acquired with fitted insoles. The participant's performance was recorded using two cameras positioned at the midpoint and terminus of the inquest route.

B. Dataset Annotation

The gait annotation concentrated on identifying the distinct events of a gait cycle: Heel strike (HES) - Foot flat (FOF) - Heel rise (HER) - Toe off (TOF) - HES. Event labeling for both legs was essential for gait analysis. In a typical gait cycle, the process commences with one foot in HES and the opposite foot in TOF. We employed a hybrid paradigm of manual and automatic labeling for data annotation. In each instance, to ensure the veracity of data labeling, cross-verifications with the signal alternation and the recorded videos were conducted. The categorization of movement phases (sitting, standing, walking, and turning) was performed solely through manual annotation based on inspection of pressure signal alternation and synchronized video recordings. Sitting and turning segments were identified using characteristic pressure patterns and body orientation changes observed in the video data and were excluded from subsequent gait cycle analysis. The automatic annotation of gait events (HES, FOF, HER, TOF) was conducted with a gait event detection system specifically created for this purpose. The algorithm's results were cross-validated utilizing the signal and video data. The gait event detection algorithm is founded on a modified core body of knowledge pertaining to state transitions and has been presented in detail [1].

1) Gait Cycle Segmentation: A crucial phase in preprocessing entailed the extraction of distinct gait cycles from the walking data. As it is shown in Fig. 2 (a), Gait cycles were delineated using heel strike (HES) events as indicators, with each cycle defined as the data occurring between two successive HES occurrences [7]. This procedure was executed for both the left and right feet. A time threshold of 0.02 seconds was implemented to eliminate noise and artifacts, hence rejecting successive HES events occurring within this interval. This guaranteed the retention of only physiologically valid events. The duration of each gait cycle was normalized to commence at zero, hence normalizing temporal alignment across all cycles irrespective of their length.

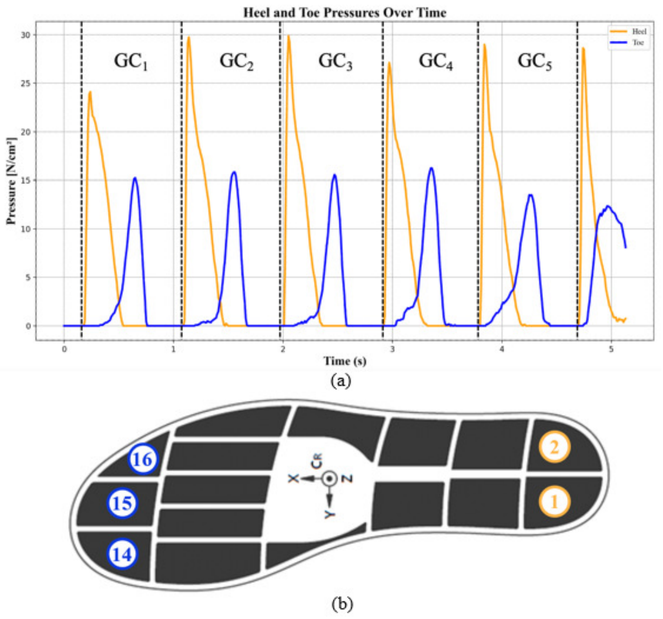

Fig. 2. (a) Indicative sample of heel and toe pressure values over time and how the gait cycles are computed, (b) Pressure sensors used in the study.

In the present work, we employed data from the signal segment associated with straight-line walking during the WST and mTUG tests, thus removing turning and sitting pressure

data. For each subject, two recordings per test were examined. Furthermore, we determined that pressure sensors 1 and 2 corresponded to the heel region, whereas sensors 14, 15, and 16 pertained to the toe region, as seen in Fig. 2 (b). The addition of extra sensors (1–4 for the heel and 9–16 for the toe) did not influence the outcomes [3].

2) Data Normalization, Filtering and Final Dataset: Several normalization and filtering procedures were implemented to prepare the data for further processing. Pressure results in each gait cycle were normalized by dividing by the cycle's maximum value. Gait cycles with durations beyond one standard deviation from the mean were eliminated to mitigate any segmentation mistakes or avoid outliers. Thus, the length of each gait cycle was determined at 1 s, i.e. 100 data points. Each gait cycle contains two channels, one comprising the pressure values for heel region and one for the toe area.

C. Conditional DC-GAN Architecture and Training

The implementation of the conditional Deep Convolutional GAN (DC-GAN) entails training the network to produce realistic synthetic datasets based on two conditional parameters: subject IDs and subject group. This dual conditional structure is intended to offer meticulous control over the data generation process, enabling the model to generate samples tailored to both subject identity and grouping.

1) Data Splitting and Leakage Prevention: To prevent data leakage, all trials adhered to a rigorous fold-wise separation method. For each cross-validation fold, the dataset was initially partitioned into training, validation, and test subsets. The conditional DC-GAN was trained solely on the training subset of each fold, utilizing authentic gait cycles corresponding exclusively to training subjects. Synthetic gait cycles were exclusively produced by this trained generator and utilized purely to enhance the training data. The validation and test sets comprised solely real gait cycles and were not subjected to the GAN at any point. This guaranteed total autonomy between training and evaluation data across the pipeline.

2) Data Preparation: The preprocessing workflow begins by reshaping each row of the original dataset from a flat 100×2 structure into a $10 \times 10 \times 2$ grid format, where each row is transformed into a 10×10 image with two channels. These channels relate to the two foot regions of interest (heel, toe). Subsequently, the image dataset is normalized to a [-1, 1] range, a standard practice in GAN training, to enhance numerical stability and performance [8]. The transformed images are subsequently shuffled and organized into a TensorFlow dataset, facilitating efficient data input during training.

3) Generator: The generator network is engineered to transform a concatenated vector of random noise and conditional inputs into realistic images of dimensions $10 \times 10 \times 2$. As shown in Fig. 3, a dense layer initiates the transformation of the input vector into a $5 \times 5 \times 128$ latent space. The latent representation is upsampled using a transposed convolution layer, each accompanied by batch normalization and Leaky ReLU activation, systematically rebuilding the spatial and channel dimensions of the output. The concluding transposed convolutional layer employs a hyperbolic tangent activation function to generate the 10×10 images with two channels.

4) Discriminator: The discriminator assesses the realism of the generated images by categorizing them as real or fake. The process begins with two convolutional layers, interspersed with Leaky ReLU activations and dropout layers to reduce overfitting. The outputs are flattened and transmitted across a dense layer, resulting in a singular scalar value that signifies the realism of the input. This architecture, presented in Fig. 3, allows the discriminator to effectively capture spatial and channel-wise correlations present in the data.

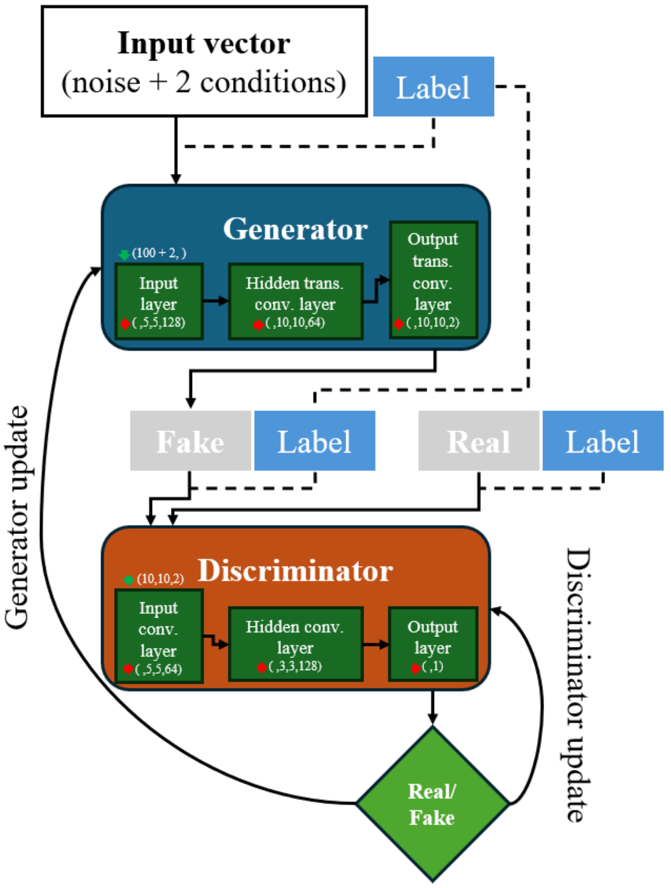

Fig. 3. Structure of the implemented conditional DC-GAN.

5) Conditions: The conditional inputs, denoting subject IDs and groupings, are incorporated into the noise vector at the generator's input (Fig. 3). Throughout the training process, these conditional vectors are randomly selected and concatenated with the noise to create an input tensor of dimensions (batch size, 102). The discriminator obtains both authentic images and their associated conditional vectors, guaranteeing congruence between the conditions and the input data. This alignment is essential for implementing conditional consistency in the produced outputs.

6) Training: The model was trained via the TensorFlow framework, where in each training step entailed supplying the generator with noise based on the desired attributes. Random noise vectors, indicative of latent space, were combined with randomly chosen condition vectors according to the batch size. The generator processed this combined input, resulting in the creation of synthetic images. The discriminator subsequently

assessed both the authentic images and the produced ones, based on the corresponding attributes, to allocate authenticity scores. Subsequently, the generator loss was obtained from the discriminator's evaluation of the synthetic images, motivating the generator to provide realistic outputs. The discriminator loss concurrently guaranteed its capacity to differentiate between real and fake images. The losses were backpropagated to adjust the weights of the generator and discriminator utilizing their respective optimizers. Both the generator and discriminator used binary cross-entropy loss and were optimized using the Adam optimizer, with a learning rate 10^{-4}.

The training loop processed all batches of the dataset for 50 epochs. During each epoch, the losses of the generator and discriminator were aggregated and averaged over batches to assess progress. Model checkpoints were established at consistent intervals, precisely every 10 epochs, to guarantee that progress could be retained and continued if required. Upon completion of the training procedure, final sample outputs were produced utilizing a predetermined seed and conditions, so establishing a reliable foundation for assessing the outcomes. This systematic technique guaranteed stability in training, harmonizing the interplay between the generator and discriminator while generating high-quality synthetic images based on the designated criteria.

7) Synthetic Gait Generation: The synthetic gait generation process utilizes the trained generative model to produce gait data based on individual subject ID and group. The procedure commences by standardizing the IDs and groups to align with the input parameters utilized during training. A random noise vector of size 100 is concatenated with the normalized conditions to create the input for the generator. For each patient, the quantity of synthetic samples produced by the generator corresponds to the quantity of original samples present in the dataset. The output images ($10 \times 10 \times 2$) are flattened into distinct arrays for heel and toe data, stored with the associated ID and group. Thus, we generate comprehensive synthetic datasets that replicate the original data distribution while integrating varied conditions.

8) Synthetic Gait Validation: The validation of synthetic data was initially conducted by calculating several statistical and distance-based metrics to assess the resemblance between synthetic and original data [9]. We computed several quantitative metrics, including Mean Absolute Error (MAE) and Mean Squared Error (MSE), which quantify the magnitude of prediction errors between estimated and true values. Furthermore, cosine similarity assessed the angular proximity of the datasets, and the Kullback-Leibler (KL) divergence and Jensen-Shannon (JS) divergence evaluated the disparities in their probability distributions. The Wasserstein Distance (WD) assessed spatial and temporal alignments, whilst the Fréchet Inception Distance (FID) measured distributional proximity in multidimensional space.

Moreover, we validated the synthetic data through the computation of gait parameters, explained in detail in our previous works [1], [3]. Metrics such as stance phase duration, swing phase duration, and foot pressure distribution elucidate the mechanical and temporal characteristics of gait. By examining the pressure-time integral and heel-to-toe ratios, we can contrast the dynamics of real and synthetic datasets, with the summed statistics indicating the mean and variability of these characteristics.

D. RPs computation

Generally, for a dynamic system, the RPs serve as a method for visualizing its recurrences [10]. The RP, in its binary form, is a matrix similar to the concept of correlation, where the "1" values signify a dynamic relationship. The purpose of the RPs is to analyze the dynamic behavior of a time series and its delayed replicas, with a delay of τ samples, whose phase space trajectories are within the same phase space, with a dimension proportional to the embedding parameter m [11]. The process seeks to identify adjacent state vectors that fundamentally reside within a specified region determined by a threshold ε. The RPs can be further examined using the recurrence quantification analysis (RQA) approach, which utilizes the binary representation of the RPs to derive metrics that quantify the intensity of the system's dynamic activity.

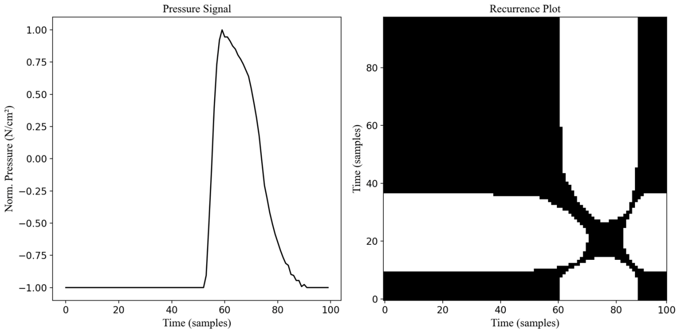

Fig. 4. Pressure values during one randomly selected gait cycle (left) and the respective RP computed (right).

Consequently, in our study, RPs were generated from the real and synthetic data to examine the dynamic behavior of heel and toe pressure signals, as seen in Fig. 4. The time series data were embedded to a higher-dimensional phase space utilizing a time delay of two and an embedding dimension of two, facilitating the reconstruction of the system's state space trajectory. The recurrence matrix was subsequently created utilizing a fixed-radius neighborhood approach with a Euclidean metric, encapsulating temporal recurrences in the pressure signals. A Theiler corrector of one was employed to mitigate the impact of short-term changes [12].

E. Vision Transformers (ViTs) for RPs Classification

This work examines multiple fusion strategies to integrate RPs from the heel and toe areas to enhance categorization utilizing ViTs. We examine four distinct methodologies, each crafted to exploit region-specific information in a complementary fashion. The four different architectures, shown in Fig. 5, are presented below:

1) Model 1: Dual ViT Stream with Late Fusion: The model comprises two autonomous ViT branches, each analyzing RP images from the toe or heel region. Each branch adheres to a conventional ViT framework, wherein images are segmented

into non-overlapping patches, linearly projected, and processed through numerous transformer layers to extract spatial-temporal characteristics. The collected feature embeddings from both branches are subsequently fused through concatenation prior to being processed by a multi-layer perceptron (MLP) head, which executes the final classification. This method enables the model to independently acquire region-specific representations while utilizing complementing information from both areas to enhance decision-making.

2) Model 2: Feature Fusion via Attention Mechanism: This model uses ViTs to analyze the RP images from the toe and heel regions, including an attention-based fusion technique. The image of each region is separately divided into patches, encoded, and processed through uniform transformer blocks to extract spatial-temporal information. Rather than merely concatenating the retrieved embeddings, an attention mechanism is employed to dynamically assess the significance of toe and heel representations. The attended features and original embeddings are flattened and concatenated to create a fused representation, which is subsequently processed by a MLP for final classification. This method allows the model to adaptively focus on the most informative areas, hence improving classification performance through the utilization of region-specific dependencies.

3) Model 3: Cross Attention Between Heel and Toe Features: In this implementation, we utilized a ViT architecture with a sophisticated attention mechanism to improve feature interaction between the two input regions. Each RP image from the toe and heel is initially segmented into patches, which are subsequently encoded via a common patch encoder. The encoded patches traverse several transformer layers to get high-level feature representations. The model employs a cross-attention method, integrating toe features as queries for heel features and vice versa, rather than simply concatenating these independent representations. This bidirectional attention allows the network to capture interdependencies between the two regions, enhancing the learned embeddings. The cross-attended features are subsequently concatenated and subjected to additional transformer layers for further refinement prior to being processed by an MLP classifier. This method guarantees that the model efficiently utilizes supplementary information from both areas, resulting in more distinct representations for categorization.

4) Model 4: Separate Models Training and Ensemble Learning: This particular method entails training two distinct ViT models—one for toe RPs and another for heel ones—prior to integrating their outputs using ensemble learning. Each model experiences patch-based tokenization, succeeded by a series of transformer layers that extract advanced features. Post-training, predictions from both models are combined utilizing majority voting, which selects the most frequently predicted class. This ensemble technique increases resilience by utilizing complementing information from both perspectives, hence enhancing classification accuracy relative to individual models.

II. DISCUSSION

The application of the conditional DC-GAN for generating gait data has yielded encouraging outcomes, underscoring the model's capacity to produce authentic gait patterns among various subject groups. The low MAE and MSE values across all groups demonstrate that the generated samples show minor discrepancies from actual data points, confirming the precision of the generative model. The close alignment in cosine similarity scores indicates that the synthetic gait cycles preserve a constant angular relationship with the original dataset, exhibiting only small differences in foot pressure dynamics, especially in the toe region for the group B (DBS). Furthermore, the probability distributions of the created dataset closely resemble those of the actual data, as demonstrated by the KL and JS divergence measures. The findings indicate that the synthetic data accurately maintains the statistical aspects of the original gait dataset, hence providing a realistic depiction of gait characteristics under various settings. Moreover, the low WD and FID scores further validate the resemblance between synthetic and real distributions, underscoring the efficacy of the DC-GAN in encapsulating critical gait characteristics. Minor differences were noted again in group B, especially regarding the pressure distribution in the toe region; nevertheless, these variations are within an acceptable range and do not substantially affect the overall validity of the obtained dataset. Interestingly, these localized divergences in the DBS group may reflect treatment-specific modulations in gait mechanics, rather than model artifacts—highlighting the potential of this approach to reveal clinically meaningful gait biomarkers

Additionally, an analysis of gait metrics between original and synthetic data uncovers nuanced but consistent patterns. The duration of the synthetic stance phase is marginally extended, resulting in a comparable reduction in swing phase duration, signifying a little temporal alteration in gait cycles. Foot pressure distribution exhibits slight fluctuations, characterized by a modest increase in heel pressure and a decrease in toe pressure. Although these changes indicate slight modifications in weight distribution, the pressure-time integral stays constant, affirming the overall stability of pressure exertion patterns. The minor rise in the heel-to-toe ratio in the generated data may suggest a slightly altered weight transition during gait; nonetheless, these variations do not substantially undermine the physiological plausibility of the synthesized gait cycles. Notwithstanding these little discrepancies, the conditional DC-GAN has effectively encapsulated the fundamental gait attributes and generated synthetic data that nearly mirrors the original dataset. The capacity to produce supplementary gait cycles while preserving class equilibrium among the three groups is very advantageous for deep learning applications, where data augmentation is essential for model generalization.

The comparative investigation of the four ViT models for multi-class and binary classification tasks reveals significant trends concerning the efficacy of various fusion procedures. In the multi-class classification challenge comprising three groups—Control, non-DBS, and DBS patients—model 1 (Dual ViT Stream with Late Fusion) attains the highest overall performance, achieving an accuracy of 94.58%, accompanied by robust precision (85.31%) and recall (83.42%). This indicates that utilizing distinct ViT streams for various input parameters and integrating them subsequently yields a strong

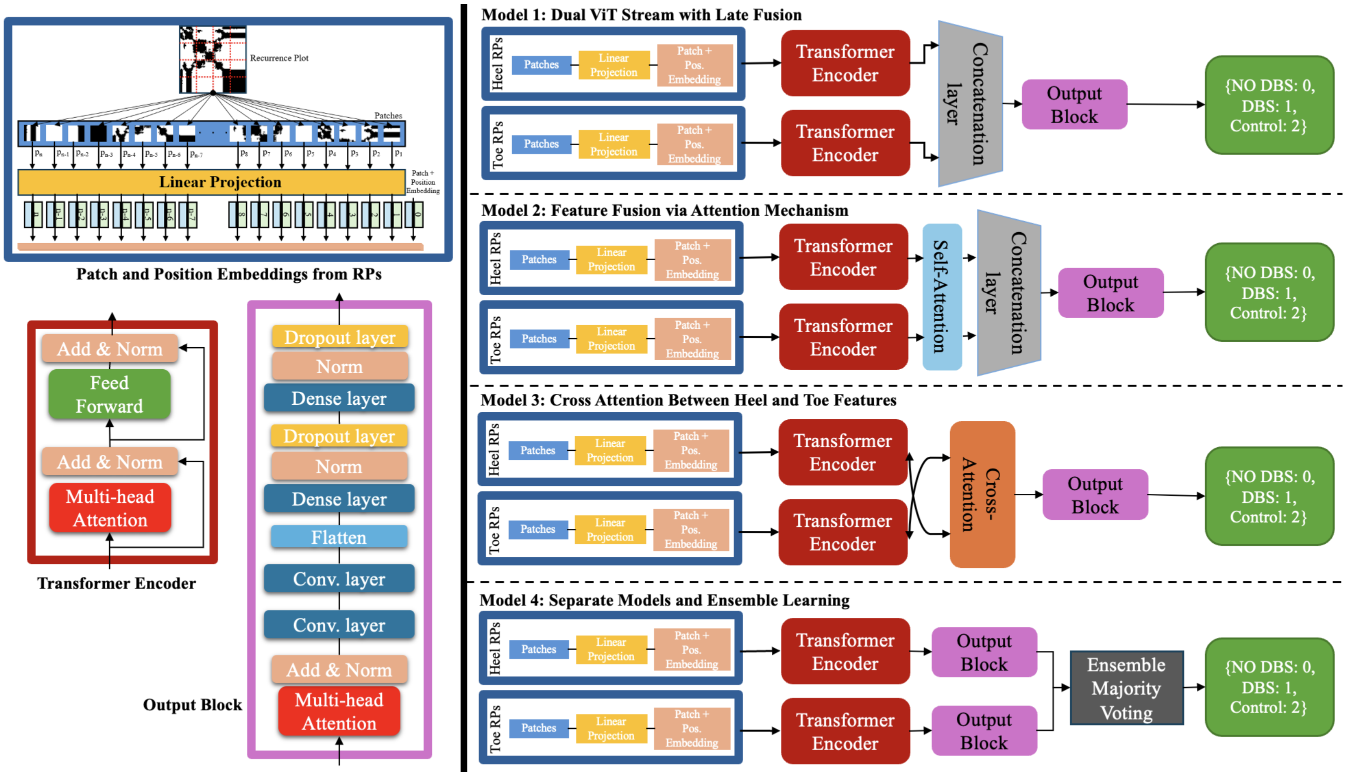

Fig. 5. Implementation of ViT models with different fusion strategies.

representation of gait characteristics among the three groups. The enhanced precision and recall metrics demonstrate that this method successfully identifies class-specific patterns, rendering it exceptionally dependable for multi-class classification. Model 2, employing attention-based feature fusion, exhibited the poorest performance, presumably due to challenges in differentiating pertinent information, potentially resulting in the introduction of noise. Model 3, utilizing cross-attention between heel and toe features, exhibited superior performance however still encountered difficulties in completely distinguishing between classes. Model 4, which utilized distinct models and ensemble learning, exhibited the lowest precision and recall, likely attributable to classification inconsistencies and difficulties in differentiating between groups.

In binary classification tasks, which include pairwise group comparisons, from a technical perspective, model 3, employing cross-attention between heel and toe features, consistently surpassed the other models. This indicates that utilizing cross-modal interactions between the heel and toe signals offers more comprehensive contextual information, hence improving the model's capacity to distinguish between patient groups. Notably, the high precision and recall in control versus DBS categorization is the main distinct point between model 3 and the rest of the models, thus highlighting the efficacy of cross-attention in discerning nuanced differences in gait patterns. Moreover, the model achieved this superior performance with the lowest computational cost, making it both effective and efficient. Additionally, model 1 exhibited robust performance in all binary classification tasks, especially in differentiating between DBS and non-DBS patients, indicating that the dual ViT stream with late fusion effectively captures gait changes potentially evoked by DBS. Nonetheless, distinguishing between control and DBS subjects was more difficult due to the convergence of gait characteristics. In a likewise manner, model 2 faced difficulties with control versus DBS categorization but excelled in detecting DBS-specific gait abnormalities, suggesting that attention-based fusion was less efficacious for general gait variations. Model 4, albeit utilizing an ensemble methodology, had inferior generalization compared to alternative fusion procedures, demonstrating diminished efficacy in differentiating controls from PD patients. This outcome further underlines that more integrated, physiologically-informed fusion strategies are preferable when modeling complex motor effects arising from therapeutic interventions.

From a clinical standpoint, the models were able to distinguish the three groups with high accuracy, suggesting that they recognize distinct gait patterns within subjects who could be considered as "similar". In clinical as well as research settings, such DL models could facilitate a refined evaluation of the influence of various treatments on gait in PD patients, thus allowing more dynamic and personalized treatment approaches. Interestingly, the model's difficulty in distinguishing particularly DBS treated patients from controls, indicates that DBS may achieve a more normal gait pattern than oral medication [13]. With the emergence of innovative DBS techniques, adjusting stimulation in real time based on fluctuating clinical needs (i.e. adaptive DBS), the development of reliable biomarkers based on wearable sensor data, is increasingly becoming more crucial.

While the proposed ViT model holds significant promise in

distinguishing patients under different treatments, based on gait features,, several limitations warrant consideration. A limitation of this study is the complexity and computational requirements of the utilized ViT models which necessitate significant computer resources for training and inference, hence complicating real-time or portable implementations. Furthermore, although the synthetic gait produced by the DC-GAN alleviates class imbalance, slight disparities persist between the original and synthetic data, particularly in the toe pressure distributions of DBS patients, potentially introducing subtle biases.

Clinically, the ability of the models to generalize across diverse populations remains uncertain, as the dataset originates from a particular cohort of PD patients in Greece. Differences in gait patterns arising from cultural or environmental influences may restrict the generalizability of the results. The latter probably explains the DBS over-repsentation in our cohort which could be expected to be less than 5% in a general PD population. However, this might counteract another possible limitation such as the individually small number of DBS patients. Moreover, patients were assessed in the ON-state, which was determined mostly temporally (about one hour after the Levodopa intake), which means that in some cases the assessments might not capture the ”optimal ON medication state” of the patients. That could be attributed to the fact that for some patients the response to levodopa was either unpredicted or did not necessarily coincide with the time frame we had set. We should also mention that for some patients with advanced PD, the ON-state came with dyskinesias, i.e. involuntary movements of the extremities and/or the trunk, which may have interpolated to produce a gait pattern that differs from the typical Parkinsonian gait. Yet, these are phenomena that may as well represent the real-world patient experience. These limitations may have affected the data and that was reflected in the suboptimal results of some models when performing the binary classifications.

References

[1] C. Chatzaki, V. Skaramagkas, N. Tachos, G. Christodoulakis, E. Maniadi, Z. Kefalopoulou, D. I. Fotiadis, and M. Tsiknakis, “The smart-insole dataset: gait analysis using wearable sensors with a focus on elderly and parkinson’s patients,” *Sensors*, vol. 21, p. 2821, Jan. 2021.

[2] R. B. Postuma, D. Berg, M. Stern, W. Poewe, C. W. Olanow, W. Oertel, J. Obeso, K. Marek, I. Litvan, A. E. Lang, G. Halliday, C. G. Goetz, T. Gasser, B. Dubois, P. Chan, B. R. Bloem, C. H. Adler, and G. Deuschl, “MDS clinical diagnostic criteria for Parkinson’s disease: MDS-PD Clinical Diagnostic Criteria,” *Movement Disorders*, vol. 30, pp. 1591–1601, Oct. 2015.

[3] C. Chatzaki, V. Skaramagkas, Z. Kefalopoulou, N. Tachos, N. Kostikis, F. Kanellos, E. Triantafyllou, E. Chroni, D. I. Fotiadis, and M. Tsiknakis, “Can gait features help in differentiating parkinson’s disease medication states and severity levels? A machine learning approach,” *Sensors*, vol. 22, p. 9937, Jan. 2022.

[4] S. A. Combs, M. D. Diehl, J. Filip, and E. Long, “Short-distance walking speed tests in people with Parkinson disease: Reliability, responsiveness, and validity,” *Gait & Posture*, vol. 39, pp. 784–788, Feb. 2014.

[5] F. Kluge, H. Gaßner, J. Hannink, C. Pasluosta, J. Klucken, and B. M. Eskofier, “Towards mobile gait analysis: concurrent validity and test-retest reliability of an inertial measurement system for the assessment of spatio-temporal gait parameters,” *Sensors*, vol. 17, p. 1522, July 2017.

[6] “Smart insoles for gait and motion analysis in research - moticon.”

[7] A. Leal-Junior and A. Frizera-Neto, “Gait analysis: overview, trends, and challenges,” in *Optical Fiber Sensors for the Next Generation of Rehabilitation Robotics* (A. Leal-Junior and A. Frizera-Neto, eds.), pp. 53–64, Academic Press, Jan. 2022.

[8] A. Radford, L. Metz, and S. Chintala, “Unsupervised representation learning with deep convolutional generative adversarial networks,” *CoRR*, vol. abs/1511.06434, 2015.

[9] A. Borji, “Pros and cons of GAN evaluation measures: New developments,” *Computer Vision and Image Understanding*, vol. 215, p. 103329, Jan. 2022.

[10] V. Skaramagkas, A. Pentari, D. I. Fotiadis, and M. Tsiknakis, “Using the recurrence plots as indicators for the recognition of Parkinson’s disease through phonemes assessment,” in *2023 45th Annual International Conference of the IEEE Engineering in Medicine & Biology Society (EMBC)*, pp. 1–4, July 2023. ISSN: 2694-0604.

[11] N. Marwan, M. Carmen Romano, M. Thiel, and J. Kurths, “Recurrence plots for the analysis of complex systems,” *Physics Reports*, vol. 438, pp. 237–329, Jan. 2007.

[12] J. Theiler, “Sensitivity of anomalous change detection to small misregistration errors,” in *Algorithms and Technologies for Multispectral, Hyperspectral, and Ultraspectral Imagery XIV*, vol. 6966, pp. 216–224, SPIE, May 2008.

[13] A. Mirelman, P. Bonato, R. Camicioli, T. D. Ellis, N. Giladi, J. L. Hamilton, C. J. Hass, J. M. Hausdorff, E. Pelosin, and Q. J. Almeida, “Gait impairments in Parkinson’s disease,” *The Lancet. Neurology*, vol. 18, pp. 697–708, July 2019.
